# Familial hypocalciuric hypercalcemia caused by homozygous *CaSR* gene mutation: A case report of a family

**DOI:** 10.1097/MD.0000000000021940

**Published:** 2020-08-28

**Authors:** Feifei Wang, Jia Hu, Chao Mei, Xia Lin, Ling Zhang

**Affiliations:** Department of Endocrinology, Huzhou First People's Hospital, The Affiliated Hospital of Huzhou Teachers College, Huzhou, Zhejiang, China.

**Keywords:** calcium-sensitive receptor, familial hypocalciuric hypercalcemia, inactivating gene mutations, parathyroid hormone

## Abstract

**Introduction:**

Familial hypocalciuric hypercalcemia (FHH) is a group of autosomal dominant genetic diseases with persistent hypercalcemia and hypocalciuria. The calcium-sensitive receptor (CaSR) plays an important role in calcium and phosphorus metabolism.

**Patient concerns:**

A 32-year-old man who had diabetes was admitted to our hospital due to poor glycemic control, and was found to have hypercalcemia, hypophosphatemia, and hyperparathyroidism. Single-Photon Emission Computed Tomography (SPECT) (99-mTcMIBI) examination result was negative. The result of 24-h urine calcium was 2.18 mmol/24 h, and the 24-h urinary calcium to creatinine ratio (UCCR) was 0.006. Family survey showed that all of the family members had hypercalcemia.

**Diagnosis:**

The *CaSR* gene mutation study revealed that the proband had a homozygous mutation for a T>C nucleotide substitution at c.1664 in exon 6, while both the mother and the father had heterozygous mutations at the same site of exon 6. The clinical diagnosis was considered to be FHH type1.

**Interventions:**

The patient was treated with conventional calcium-lowering therapy which was not effective. Cinacalcet was suggested but not used. The patient received salmon calcitonin nasal spray and furosemide tablets treatment for 1 month after discharge, and then stopped the medication.

**Outcomes:**

On follow up 4 months after being discharged, the serum calcium level was 3.18 mmol/L, and the PTH level was 275.4 ng/mL. He had felt fatigued, intermittent abdominal pain and lost 3.9 kg of weight.

**Conclusion:**

This case studied a family with FHH, and the *CaSR* gene c.1664T>c mutation was the possible pathogenic cause. If parathyroid location examination is unclear for hyperparathyroidism, the possibility of FHH should be considered. For FHH patients, conventional calcium reduction therapy was ineffective and parathyroid surgery cannot alleviate their hypercalcemia.

## 1 Introduction

Familial hypocalciuric hypercalcemia (FHH) is a group of autosomal dominant genetic diseases, which is characterized by persistent hypercalcemia, hypophosphatemia, hypermagnesemia, normal or mildly elevated serum parathyroid hormone (PTH) levels and low urinary calcium excretion.^[1]^

Calcium-sensitive receptor (CaSR) and its signal transduction play an important role in the regulation of extracellular calcium homeostasis. The *CaSR* gene is located at 3q13.3–21 and contains 8 exons encoding a 1078 amino acid which belongs to class C of G protein-coupled receptor superfamily.^[2]^ The signal transduction pathway of CaSR also includes the G-protein α-11 (Gα_11_) subunit and the adapter-related protein complex 2σ (AP2σ) subunit, encoded by the *GNA11* and *AP2S1* genes, respectively. CaSR can recognize small changes in extracellular Ca^2+^ and maintains calcium homeostasis in the body by regulating the synthesis and secretion of PTH and the urinary calcium reabsorption.^[3]^ At present, more than 230 different *CaSR* gene mutations have been reported, of which more than 130 are inactivated mutations leading to dysfunction of CaSR.^[4]^ The inactivating mutations of the *CaSR* gene cause an elevated calcium setting point that results in an inappropriate secretion of PTH and an increase in renal tubular reabsorption of Ca^2+^.

Here, we present the case of a 32-year-old patient who was diagnosed with diabetes at the age of 14. The patient was found to have hypercalcemia during the treatment of diabetes. Further studies demonstrated that the patient possessed the c.1664T>C homozygous mutation of *CaSR* gene. We further investigated the patient's family history. The study was approved by the ethics committee of Huzhou first people's hospital. The patient and his family all provided informed consent for publication of this report.

## 2 Patient information and clinical findings

In March 2017, a 32-year-old man who had a history of diabetes for more than 10 years was hospitalized in the department of endocrinology due to poor glycemic control. He had been using insulin Novolin 30R since his diagnosis. The patient had cognitive and motor development retardation from infancy. There was no abnormality in maternal pregnancy history and productive history. The patient and his family denied the history of hypercalcemia at that time. The physical examination revealed that the patient had a height of 162 cm, a bodyweight of 46.9 kg, partial tooth loss, and scoliosis. We rated the patient using the Mini-Mental Status Examination (MMSE) and scored 8 points.

Initial laboratory evaluation revealed hypercalcemia (serum calcium 3.69 mmol/L, RR 2.0–2.6 mmol/L), hypophosphatemia (serum phosphorus 0.40 mmol/L, RR 0.67–1.04 mmol/L), and hyperparathyroidism (PTH 284.0 pg/mL, RR 15–68.3 pg/mL). Alkaline phosphatase (ALP) level, growth hormone level, reproductive hormone levels, markers of thyroid function and adrenal function were all normal. The glucose tolerance test indicated that the blood glucose level was 9.34 to 10.12 to 12.7 to 16.3 to 15.07 mmol/L, and the C peptide level was 0.6 to 1.0 to 1.3 to 1.7 to 1.5 ng/mL.

**Figure 1:**
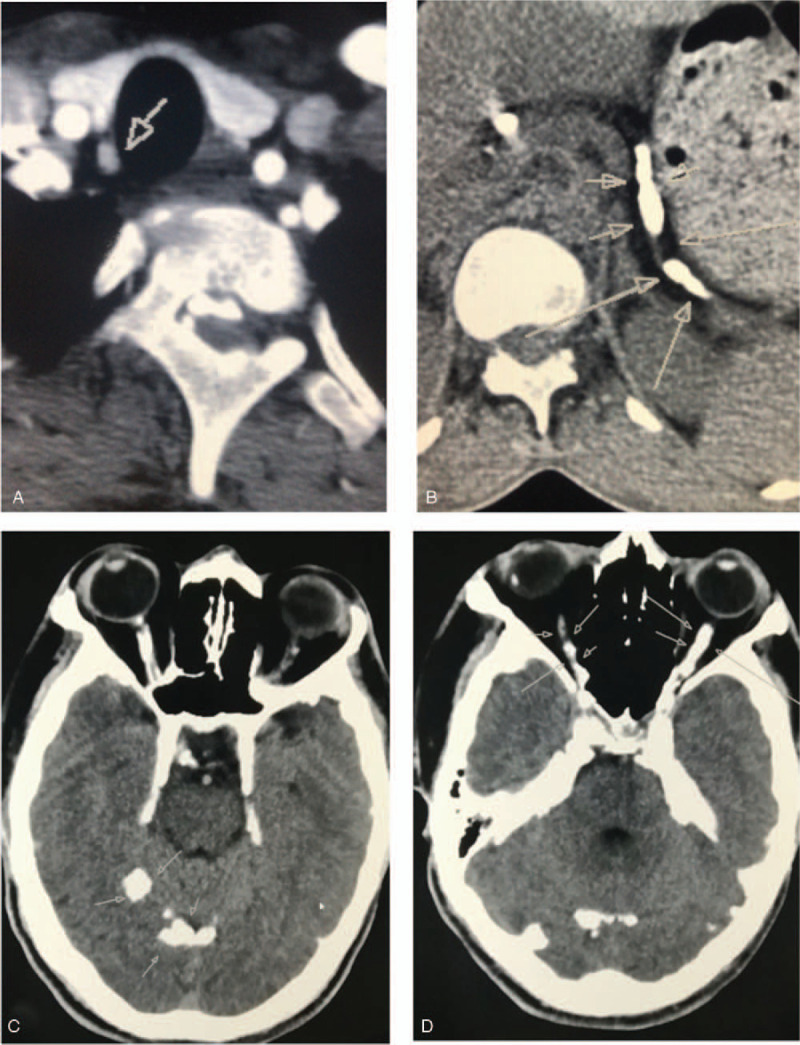
Some of the proband's radiograph findings. (A) Thyroid-enhanced CT suggested a suspicious nodule below the right thyroid gland; (B) Pancreas-enhanced CT suggested pancreatic atrophy and multiple stones in the pancreatic duct; (C and D) Head CT suggested extensive meningeal calcification including tentorium cerebelli, cerebral falx, and bilateral optic nerve calcification.

X-ray of hand, ulna, humerus, pelvis, and chest showed no obvious abnormalities, as well as the cardiac color Doppler ultrasound. Abdominal computed tomography (CT) showed pancreatic atrophy with calcification. Bone mineral density test suggested that the bone density scores of the lumbar spine and hip joint were lower than those of patients of the same sex and age. Thyroid-enhanced CT suggested a suspicious nodule below the right thyroid gland (Fig. 1A). Parathyroid Single-Photon Emission Computed Tomography (SPECT) (99-mTcMIBI) examination suggested that no abnormal radioactive aggregation was observed in the parathyroid imaging.

In October 2018, the patient was admitted to the department of surgery due to abdominal pain. Laboratory findings at admission showed serum calcium of 3.29 mmol/L, serum magnesium of 1.13 mmol/L (RR 0.67–1.04 mmol/L), serum phosphorus of 0.57 mmol/L, PTH level of 197.8 pg/mL, but continuous normal serum amylase. Pancreas-enhanced CT suggested pancreatic atrophy and multiple stones in the pancreatic duct (Fig. 1B), and magnetic resonance cholangiopancreatography (MRCP) suggested pancreatic duct dilatation with stone formation.

In January 2019, the patient was hospitalized in the department of endocrinology again. In the past 1 year, he had suffered from chest tightness, fatigue, and intermittent abdominal pain. He was limited by the stairs and required a handrail. This time, laboratory testing revealed that serum calcium was 3.76 mmol/L, serum magnesium was 1.14 mmol/L, serum phosphorus was 0.66 mmol/L, and PTH was 666.1 pg/mL. The 24-h urine calcium was 2.18 mmol/24 h, and the 24-h urinary calcium to creatinine ratio (UCCR) was 0.006. Head CT suggested extensive meningeal calcification including tentorium cerebelli, cerebral falx, bilateral optic nerve calcification, and strip calcification under both sides of the skull (Fig. 1C and D). Repeat thyroid-enhanced CT was similar to that of 2017 (Fig. 1A).

## 3 Family survey

The patient has one elder brother, and their parents are closely related cousins. His brother was found to have hypercalcemia during a physical examination in June 2017. He had experienced chest tightness and fatigue symptoms since May 2018 and underwent “left parathyroidectomy” in another hospital on November 20, 2018. The brother's preoperative levels of serum calcium and PTH were 2.84 mmol/L and 88.3 pg/mL, respectively. Postoperative serum calcium and PTH levels were 2.65 mmol/L and 46.8 ug/mL, respectively, and later review were 2.7 to 2.84 mmol/L and 79 to 114 ug/mL, respectively.

Their mother was found to have hypercalcemia in March 2018 as well, and underwent “left parathyroidectomy” in December 2018. Her preoperative levels of serum calcium and PTH were 2.95 mmol/L and 79.2 pg/mL, respectively. Postoperative serum calcium and PTH levels were 2.53 mmol/L and 60.3 ug/mL, respectively, and later review was 2.6 to 2.8 mmol/L and 99 to 112 ug/mL, respectively.

Their father denied a similar history of hypercalcemia. Electrolyte and PTH levels of the parents, brother, and other three family members were examined on January 11, 2019, and the results are shown in Table 1.

**Table 1:**
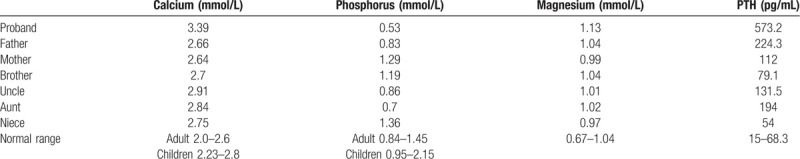
Electrolyte and PTH levels of family members.

## 4 Genetic evaluation

After the informed consent was obtained, we extracted whole blood samples of the proband and his family members and sent them to Beijing MyGenostics medical laboratory for DNA testing. NextSeq500 system high throughout benchtop (Illumina, USA) was used for full exon sequencing after obtaining the target gene. Sanger sequencing was used to investigate the mutation of CaSR.

**Figure 2:**
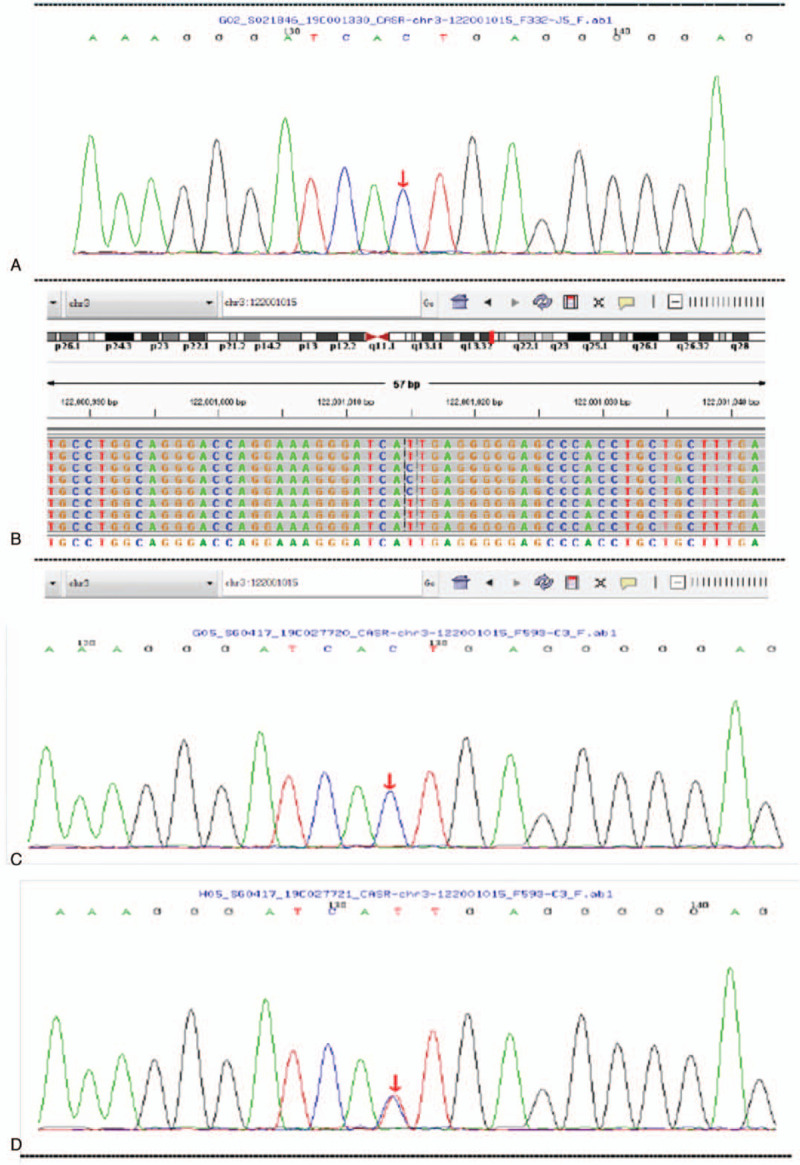
The gene test results. (A) The proband had a homozygous mutation for a T>C nucleotide substitution at c.1664 in exon 6; (B) Both the mother and father had heterozygous mutations at the same site of exon 6; (C) The patient's brother and aunt have homozygous variants at c.1664 in exon 6; (D) The patient's uncle and niece have heterozygous variants c.1664 in exon 6.

**Figure 3:**
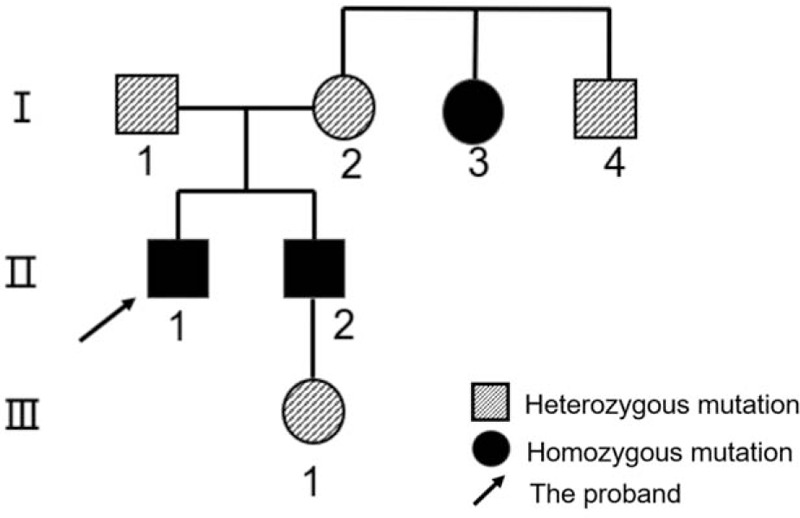
Pedigree of the family under study. Circles indicate female and squares indicate male.

The *CaSR* gene mutation study of the proband revealed that there was a homozygous mutation for a T>C nucleotide substitution at c.1664 in exon 6, which resulted in an amino acid change I555T (replaced an isoleucine with a threonine) in the extracellular domain of CaSR protein. CaSR mutation test of the patient's parents showed that both of them had heterozygous mutations at the same site of exon 6 (Fig. 2). The patient's brother and aunt had homozygous variants at this site, whereas the patient's uncle and niece had heterozygous variants at this site. The clinical diagnosis was considered to be FHH1. The pedigree of the family for CaSR is shown in Figure 3.

## 5 Interventions and follow up

In terms of diabetes, insulin Aspart 30 injection treatment showed good blood glucose control. During three times of hospitalizations, the patient had been treated with salmon calcitonin injection (50 u every 12 h), furosemide injection (20–40 mg every day), zoledronic acid injection (5 mg, November 18, 2018), and fluid replacement, but the lowest serum calcium was 3.26 mmol/L. Due to economic reasons, the patient and his family refused to undergo plasma exchange or hemodialysis treatment, and did not accept cinacalcet treatment.

After being discharged on January 21, 2019, the patient received salmon calcitonin nasal spray (200 iu twice a day) and furosemide tablets treatment (20 mg/day) for 1 month. Then, the medicine treatment was stopped. On follow up 4 months after discharge, the serum calcium level was 3.18 mmol/L, and the PTH level was 275.4 ng/mL. During this period, he felt fatigued, intermittent abdominal pain and lost his weight of 3.9 kg.

## 6 Discussion

FHH is comprised of three genetically distinct conditions, which are designated as FHH type 1 to 3. *GNA11* gene on chromosome 19p and *AP2S1* gene on chromosome 19q13.3 are considered to be responsible for FHH2 and FHH3, respectively.^[5,6]^ Patients with FHH1 are usually asymptomatic and may occasionally be diagnosed during physical examination. A few patients may have non-specific symptoms such as mild muscle weakness and fatigue, and a few cases may have clinical manifestations such as pancreatitis and chondrocalcinosis. Compared with FHH1, FHH3 is associated with a more severe biochemical phenotype. Patients with FHH3 usually show significantly higher serum calcium and magnesium, higher PTH levels, and lower urinary calcium excretion, and more obvious clinical symptoms, which can be combined with cognitive impairment and psychomotor dysplasia.^[5,7]^ The clinical manifestations of the proband in this study were more similar to FHH3, but no mutations in *GNA11* and *AP2S1* genes were detected. The clinical diagnosis was considered to be FHH1. This case demonstrated the heterogeneity and particularity of the clinical manifestations of FHH.

The biochemical features of FHH are similar to those of primary hyperparathyroidism (PHPT), but parathyroidectomy cannot change the abnormal serum calcium setting point caused by gene mutations. PHPT patients usually present with hypercalcemia, hypophosphatemia, high PTH, and hyper ALP. The pathological types of PHPT include parathyroid adenoma, polyglandular hyperplasia, and parathyroid adenocarcinoma. However, FHH patients often present with mild parathyroid hyperplasia, which is generally negative on locational examination. In addition, FHH is usually free of renal and skeletal lesions.^[8]^ Of course, the most important distinction is the urinary calcium excretion, and UCCR of FHH patients are often <0.01, while PHPT patients are often accompanied by elevated urinary calcium.^[9]^ Unfortunately, we have not examined the 24-h UCCR of family members due to certain restrictions.

*CaSR* gene inactivation mutation can cause FHH1 and neonatal severe hyperparathyroidism (NSHPT). In general, FHH1 is due to the heterozygous mutation of CaSR, while NSHPT is caused by the homozygous mutation. However, it has been reported that homozygous mutations of CaSR can also cause FHH1, and a few severe heterozygous inactivating mutations can also lead to NSHPT, which may be related to the degree of influence of gene mutations on protein function at different sites.^[10,11]^ A case of NSHPT with the heterozygous mutations for a T>C nucleotide substitution at the same site as the proband of our study has been reported.^[12]^ Calcimimetic agents such as cinacalcet can effectively alleviate hypercalcemia and high serum PTH, which are considered to be potential targeted therapy for NSHPT and FHH.^[4]^ It is recommended to have partial parathyroidectomy when the patient also has chronic pancreatitis, although there may be persistent hypercalcemia after surgery.^[13]^ The proband in this study had a long history of diabetes for more than 10 years, but no family history of diabetes, and had decreased islet function but not failure. We considered that long-term hypercalcemia would lead to calcification and atrophy of the pancreas, thus leading to decreased islet function and pancreatic diabetes. We need more evidence to prove it.

In this study, we studied a family with FHH, and the *CaSR* gene c.1664T> c mutation was the possible pathogenic cause behind the FHH of this family. This case reports homozygous mutations of CaSR causing FHH, which demonstrates that clinical phenotypes are associated with the severity of functional impairment. It is possible that long-term hypercalcemia caused pancreatic diabetes in the proband. For FHH patients, conventional calcium reduction therapy is ineffective and parathyroid surgery cannot alleviate their hypercalcemia. If parathyroid location examination is unclear for hyperparathyroidism, the possibility of FHH should be considered.

## Author contributions

**Data curation:** Xia Lin.

**Formal analysis:** Feifei Wang.

**Funding acquisition:** Feifei Wang, Jia Hu, Ling Zhang.

**Investigation:** Feifei Wang, Jia Hu, Chao Mei.

**Methodology:** Chao Mei, Xia Lin.

**Project administration:** Ling Zhang.

**Supervision:** Xia Lin.

**Validation:** Ling Zhang.

**Writing – original draft:** Feifei Wang.

**Writing – review & editing:** Jia Hu.
